# Sphingosine‐1‐Phosphate Modulates the Effect of Estrogen in Human Osteoblasts

**DOI:** 10.1002/jbm4.10037

**Published:** 2018-03-15

**Authors:** Duangrat Tantikanlayaporn, Irina L Tourkova, Quitterie Larrouture, Jianhua Luo, Pawinee Piyachaturawat, Michelle R Witt, Harry C Blair, Lisa J Robinson

**Affiliations:** ^1^ Division of Cell Biology Faculty of Medicine Thammasat University Pathumthani Thailand; ^2^ Veterans Affairs Medical Center Pittsburgh PA USA; ^3^ Department of Pathology University of Pittsburgh Pittsburgh PA USA; ^4^ Department of Physiology, Faculty of Science Mahidol University Bangkok Thailand; ^5^ Departments of Pathology and of Microbiology Immunology, and Cell Biology, West Virginia University School of Medicine Morgantown WV USA

**Keywords:** BONE MATRIX, ESTROGENS, MATRIX MINERALIZATION, MOLECULAR PATHWAYS, OSTEOBLASTS

## Abstract

Production of sphingosine‐1‐phosphate (S1P) is linked to 17β‐estradiol (E2) activity in many estrogen‐responsive cells; in bone development, the role of S1P is unclear. We studied effects of S1P on proliferation and differentiation of human osteoblasts (hOB). Ten nM E2, 1 μM S1P, or 1 μM of the S1P receptor 1 (S1PR1) agonist SEW2871 increased hOB proliferation at 24 hours. S1PR 1, 2, and 3 mRNAs are expressed by hOB but not S1PR4 or S1PR5. Expression of S1PR2 was increased at 7 and 14 days of differentiation, in correspondence with osteoblast‐related mRNAs. Expression of S1PR1 was increased by E2 or S1P in proliferating hOB, whereas S1PR2 mRNA was unaffected in proliferating cells; S1PR3 was not affected by E2 or S1P. Inhibiting sphingosine kinase (SPHK) activity with sphingosine kinase inhibitor (Ski) greatly reduced the E2 proliferative effect. Both E2 and S1P increased SPHK mRNA at 24 hours in hOB. S1P promoted osteoblast proliferation via activating MAP kinase activity. Either E2 or S1P increased S1P synthesis in a fluorescent S1P assay. Interaction of E2 and S1P signaling was indicated by upregulation of E2 receptor mRNA after S1P treatment. E2 and S1P also promoted alkaline phosphatase expression. During osteoblast differentiation, S1P increased bone‐specific mRNAs, similarly to the effects of E2. However, E2 and S1P showed differences in the activation of some osteoblast pathways. Pathway analysis by gene expression arrays was consistent with regulation of pathways of osteoblast differentiation; collagen and cell adhesion proteins centered on Rho/Rac small GTPase signaling and Map kinase or signal transducer and activator of transcription (Stat) intermediates. Transcriptional activation also included significant increases in superoxide dismutase 1 and 2 transcription by either S1P or E2. We demonstrate that the SPHK system is a co‐mediator for osteoblast proliferation and differentiation, which is mainly, but not entirely, complementary to E2, whose effects are mediated by S1PR1 and S1PR2. © 2018 The Authors *JBMR Plus* is published by Wiley Periodicals, Inc. on behalf of American Society for Bone and Mineral Research.

## Introduction

Balanced bone formation by osteoblasts and bone resorption by osteoclasts is required to maintain bone mass. Osteoblasts are regulated by a number of cytokines and growth factors, including the lipid‐derived secondary extracellular signaling molecule sphingosine‐1‐phosphate (S1P). In bone, S1P is produced both by osteoblasts^1^ and osteoclasts.^2^ Circulating S1P in humans is associated with a negative effect on bone mass,^3^ although S1P in osteoblasts promotes growth and survival.^4^ Estrogen modulates S1P production in cells including astroglia, breast, and endothelium.^5^, ^6^, ^7^ In bone, estrogen is essential for skeletal development and maintenance with a mixture of direct and indirect effects on growth and on the balance of bone formation and resorption.^8^ The relation of estrogen and S1P signaling in bone is poorly studied.

S1P is synthesized from sphingosine by sphingosine kinases (SPHK), and it acts by G‐protein coupled receptors (S1PRs). S1P modifies proliferation, differentiation, migration, survival, and calcium homeostasis in a variety of cells by coupling receptor of activation to a variety of downstream activators including Map kinases and transcription factors.^9^ S1P is irreversibly degraded by S1P lyase to palmitaldehyde, and its phosphorylation is reversed by phosphatases.^10^ Several plasma membrane sphingosine‐1 phosphate receptors^11^ mediate pathophysiological effects of S1P; because of specific links of individual receptors to effects, receptor‐specific inhibitors are potential therapeutic agents.^12^

We investigated the effect of estradiol and S1P in human osteoblast precursors, including on osteoblast differentiation, osteoblast production of S1P, and S1P signaling in osteoblasts. Effects of S1P on osteoblast proliferation and differentiation were examined. Molecular Pathway analysis using Affymetrix arrays to study whole‐genome mRNA expression showed that S1P and estradiol have many similar effects on osteoblast proliferation and differentiation, although differences were revealed by study of effects on individual pathways including RANKL/osteoprotegerin signaling.

## Materials and Methods

### Chemicals

17‐β‐Estradiol (estradiol) was from Sigma‐Aldrich (St. Louis, MO, USA). *D‐erythro*‐sphingosine‐1‐phosphate (S1P) was from Avanti Polar Lipids (Alabaster, AL, USA). The S1P receptor‐1 (S1PR1) agonist SEW2871 was from Tocris (Bristol, UK). The sphingosine kinase inhibitor 5‐(2‐Naphthalenyl)‐1H‐pyrazole‐3‐carboxylic acid 2‐[(2‐hydroxy‐1‐naphthalenyl) methylene] hydrazide (SKi) was from EMD Chemical (Gibbstown, NJ, USA).

### Cells

Normal human osteoblasts, CC‐2538, and normal human bone marrow‐derived mesenchymal stem cells, PT‐2501 (Lonza, Allendale, NJ, USA), were used, grown in medium containing fetal bovine serum and gentamicin and amphotericin‐B. For differentiation, 10 mM glycerol‐2‐phosphate, 50 μg/mL ascorbic acid (Lonza), 2 mM CaCl_2_, and 10 nM 1,25‐dihydroxy vitamin D_3_ were added. Charcoal‐stripped fetal bovine serum was used to eliminate endogenous lipids. Cells were grown at 37°C with 5% CO_2_. At 80% confluence, cells were detached with 0.05% trypsin in 0.02% EDTA. All experiments were performed after the third passage. Media were replaced at 2‐ to 3‐day intervals.

### Cell proliferation

Cells were plated at 9000/cm^2^. Proliferation was estimated by colorimetric MTT (3‐[4, 5‐dimethylthiazol‐2‐yl]‐2, 5‐diphenyltetrazolium bromide) assay. The reaction product, a blue formazan derivative, was measured photometrically after MTT, 0.5 mg/mL, was added to each well and incubated at 37°C for 4 hours. Dimethylsulfoxide was added to each well to solubilize the formazan crystals. Microtiter plates were placed on a shaker for 5 minutes, and absorbance was measured at 570 nm. Regarding use of the MTT assay, we performed signaling pathway analysis by MetaCore using whole‐gene expression data from 3 × 10^6^ each of control, S1P, and estrogen‐treated cells by Affymetrics chip analysis. NAD(P)H‐dependent cellular oxidoreductase enzymes were not affected by S1P and estrogen treatment. This indicates that difference of MTT assay data reflect the number of viable cells rather than altered metabolism.

### Alkaline phosphatase activity and alizarin red assay

Alkaline phosphatase activity was determined using a phosphatase substrate and a diazonium salt to substrate degradation as blue color. Medium was removed; cells were washed with phosphate‐buffered saline and fixed with 60% acetone‐citrate for 30 seconds, then rinsed with deionized water. Substrate was 0.1 mg/mL napthol AS‐MX phosphate in buffer at pH 8. The diazonium salt was fast violet, 0.6 mg/mL. The reaction was stopped by rinsing with water. Quantitative ALP activity was quantified by densitometry analysis and normalized to cell number to confirm the in situ labeling.

For alizarin red staining, cells were fixed in 70% ethanol and stained with 1% alizarin red for 2 minutes. Cells were then washed with distilled water and viewed under the light microscope.

### RNA and reverse transcription

Total RNA was prepared by phenol/guanosine isothicyanate extraction (Trizol, Invitrogen, Carlsbad, CA, USA). RNAse inhibitor (RNaseOUT, Invitrogen) was added for stability. RNA was determined by absorbance at 260 nm. Reverse transcription used 500 ng of total RNA, random hexamer primers, and Moloney murine leukemia virus reverse transcriptase (Superscript III; Invitrogen).

### Real‐time PCR

Quantitative polymerase chain reaction was as described^13^ using brilliant SYBRIII green (Stratagene, Agilent Technologies, Santa Clara, CA, USA). A mixture with nucleotides and buffer was used, adding 2.5 mM Mg, 100 nM oligonucleotide primers, and first‐strand cDNA. After 10 minutes at 95°C, cycles of 15 seconds at 95°C and 1 minute at 60°C were done. Messenger RNA between samples was calculated using the comparative cycle threshold (CT) method (ΔCT), using as control glyceraldehyde‐3‐phosphate dehydrogenase (GAPDH) mRNA. Sequences of primers for PCR are shown in Table 1.

**Table 1:** Primers for PCR

Gene	Sequence	Amplicon (bp)
*GAPDH*	F‐GAGTCAACGGATTTGGTCGT	238
	R‐TTGATTTTGGAGGGATCTCG	
*RUNX2*	F‐CCTCGGAGAGGTACCAGATG	247
	R‐TTCCCGAGGTCCATCTACTG	
*OSX (SP7)*	F‐GCCAGAAGCTGTGAAACCTC	161
	R‐GCTGCAAGCTCTCCATAACC	
*ALP*	F‐CCTTGCTCACTCACTCACTCC	182
	R‐TTTTTTTTGCCGTTCCAAAC	
*COL1A1*	F‐AGGGCCAAGACGAAGACATCCC	108
	R‐TGTCGCAGACGCAGATCCG	
*OCN (BGLAP)*	F‐GTGCAGAGTCCAGCAAAGGT	152
	R‐TCAGCCAACTCGTCACAGTC	
*OPG*	F‐AACGGCAACACAGCTCACAAGAAC	160
	R‐TGCTCGAAGGTGAGGTTAGCATGT	
*RANKL*	F‐ATCGTTGGATCACAGCACATC	152
	R‐AGACTCACTTTATGGGAACCAGA	
*SPHK1*	F‐AGGCTGAAATCTCCTTCACGC	113
	R‐GTCTCCAGACATGACCACCAG	
*S1PR1*	F‐ATATCAGCGCGGACAAGGAG	136
	R‐CACTTGCAGCAGGACATGATCC	
*S1PR2*	F‐TTCGTGCGACTACAGAACCACTGT	113
	R‐AGAAGTCGTCAAGTGGCAGCTGAT	
*S1PR3*	F‐CGGCATCGCTTACAAGGTCAA	99
	R‐GCCACGAACATACTGCCCT	
*S1PR4*	F‐GACGCTGGGTCTACTATTGCC	135
	R‐CCTCCCGTAGGAACCACTG	
*S1PR5*	F‐GCGCACCTGTCCTGTACTC	94
	R‐GTTGGTGAGCGTGTAGATGATG	
*SGPP1*	F‐CTGGTGTTCTCTAGTTTGCCTAAG	136
	R‐GGTTGAAGTTGTCAATCAGGTCC	
*ENPP1*	F‐TTGGACCCTCAGTGGCAACTTG	136

### Genomewide expression screening

Osteoblast mRNA from 3 × 10^6^ each of control cells or after 24‐hour treatments with 1 μM S1P, or 10 nM E2, was used to make biotin‐labeled cRNA and hybridized to Affymetrix (Santa Clara, CA, USA) arrays as described.^14^ These are DNA arrays on glass, using the Hu 133.2 probe‐set of 54675 oligo‐DNAs, with 20 replicates per target. Segments of most genes, typically two or more probes per gene, are included. Presence of transcripts and differences between treatments were determined from the signal and variation of each assay replicate, with statistical confidence indicated. Analysis included determination of effects on common metabolic pathways by MetaCore (St. Joseph, MI, USA) comparing effects of S1P and estrogen treatment and control against a library of 121 intracellular pathways. Analysis excluded genes not expressed with *p* > 0.05. Differences with *p* < 0.002 between conditions were included in analysis.

### Cell lysates, Western blots, and antibodies

Cells were rinsed with cold PBS and lysed on ice in 150 mM NaCl, 10 mM EDTA, 50 mM Tris, 1 mM EDTA, 1 mM orthovanadate, 15 mM NaF, 1% Triton X‐100, and 10% glycerol, pH 7.4, plus phosphatase and proteinase inhibitors, for 10 minutes; lysates were centrifuged to remove cell debris. Protein was determined using the bicinchoninic acid (BCA) method. For Western blotting, lysates were denatured in Laemmli sample buffer by boiling, separated by SDS‐polyacrylamide gel electrophoresis, and transferred to polyvinylidene difluoride derivatized nylon. Membranes were blocked in 5% BSA or milk in tris‐buffered saline‐Tween 20, 1 hour, 20°C and washed. Primary antibody was added overnight at 4°C. Rabbit monoclonal, anti‐phospho‐p44/42 MAPK (ERK1/2), anti‐p44/42 MAPK (ERK1/2), anti‐phospho‐AKT, or anti‐AKT (Cell Signaling, Danvers, MA, USA) were used at 1:1000; rabbit polyclonal anti‐S1PR1 and goat polyclonal anti‐S1PR2 (Novus Biologicals, Littleton, CO, USA) were used at 1 μg/mL; mouse monoclonal anti‐actin (Sigma‐Aldrich) was used at 1:10000. After washing, secondary antibodies at 1:20,000 were added, 1 hour, 20°C (anti‐rabbit IgG, anti‐goat IgG, and anti‐mouse IgG, Jackson ImmunoResearch, West Grove, PA, USA). Proteins were visualized by chemiluminescence (visualized by enhanced chemiluminescence (Super Signal West Femto Maximum, Thermo Fisher Scientific, Waltham, MA, USA). Blots were reprobed after membranes were stripped in Restore Plus (Thermo Fisher Scientific).

### Fluorescent assay for S1P production

Production of S1P was measured fluorometrically as described.^15^ Briefly, 1 μg of mouse monoclonal SPHK1 antibody was added to cell lysates incubated overnight at 4°C. Then 30 µL of protein A/G agarose (Santa Cruz Biotechnology, Dallas, TX, USA) was added at 4°C for 1 hour. Antibody precipitated proteins were collected by centrifugation. Pellets were washed, and 50 mM HEPES, 30 mM MgCl_2_, 0.1% Triton X‐100, NaF, and 2 mM ATP, pH 7.5, was added. Fifty microliters of this mixture was incubated with 50 µM NBD‐sphingosine for 30 minutes at room temperature; 100 µL potassium phosphate, pH 8.5, was added, followed by chloroform/methanol extraction (500 µL, 2:1) with recovery of the NBD‐S1P in the aqueous phase. The aqueous layer was analyzed for NBD‐S1P‐fluorescence with excitation at 485 nm and emission at 538 nm. A reaction without SPHK served as blank. Activity is expressed as pmole/minute/mg protein.

### Statistics

Significance of paired of results was determined with Student's *t* test using Prism 5.0 (GraphPad, La Jolla, CA, USA). Data represent at least two independent experiments (that is, separate cell cultures) with two to four replicates from each experiment. Differences were considered significant at *p* < 0.05. Analysis of variances and the Newman‐Keuls test were used for multiple comparisons.

## Results

### Estradiol or sphingosine‐1‐phosphate increase human osteoblast proliferation at 24 hours

Active proliferating cells were evaluated using the MTT assay. Regarding use of the MTT assay, we performed signaling pathway analysis by MetaCore using whole‐gene expression data from 3 × 10^6^ each of control, S1P, and estrogen‐treated cells by Affymetrics chip analysis. NAD(P)H‐dependent cellular oxidoreductase enzymes were not affected by S1P and estrogen treatment. This indicates that difference of MTT assay data reflect the number of viable cells rather than altered metabolism.

**Figure 1:**
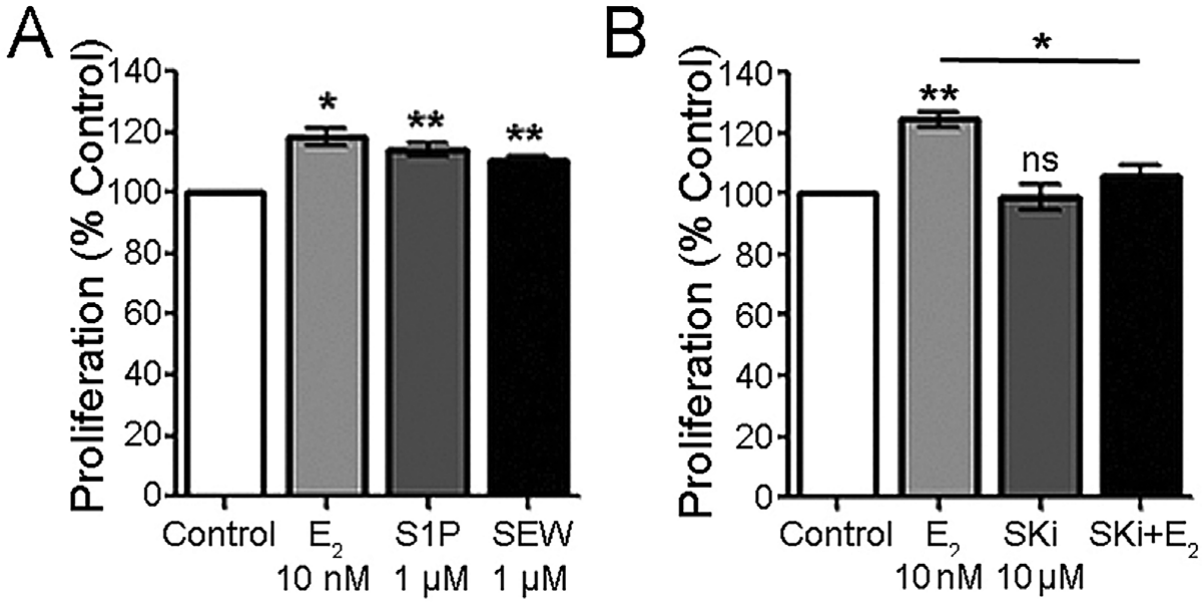
Effect of estrogen (E2) and sphingosine‐1‐phosphate (S1P) on human osteoblast cell growth at 24 hours. (*A*) Effect of estradiol, S1P, and an S1PR agonist on osteoblast cell proliferation. At 24 hours, estradiol (10 nM) increased proliferation 20% relative to control. S1P (1 μM) or SEW2871 (1 μM) induced cell growth but less than estradiol, 14% and 10%, respectively. Mean ± SEM, *n* = 6.*p < 0.05, **p < 0.01 versus control. (*B*) Sphingosine kinase inhibitor (SKi) reduces the estradiol effect on proliferation. SKi alone did not affect proliferation (second bar); SKi with estrogen reduced the effect of estrogen on cell proliferation by two‐thirds. Mean ± SEM, *n* = 4. **p < 0.01, ns (not significant), versus control; *p < 0.05 relative to estrogen only.

In Fig. 1 *A*, after exposure to 10 nM estradiol (E2) for 24 hours, osteoblast proliferation increased 20% relative to the control (*p* < 0.01). MTT also showed increases with sphingosine‐1‐phosphate (S1P) (1 μM), or with the sphingosine‐1‐phosphate receptor‐1 agonist SEW2871, by 10% to 14% (*p* < 0.05) at 24 hours. This is potentially important because of the receptor specificity of this agonist.^16^

Further to investigate the involvement of sphingosine kinase (SK) on the estradiol effect, hOB were treated with a nonspecific sphingosine kinase inhibitor (SKi), with and without E2. SKi inhibits both SPHK1 and SPHK2,^17^ so this agent does not differentiate which sphingosine kinases are active. However, SPHK1 was expressed strongly in hOB; SPHK2 was not detected in several assays. SKi alone did not affect the growth of cells; however, it reduced the effect of E2 by about 80% in the co‐treatment (Fig. 1 *B*), consistent with a role for sphingosine kinase in the effect of estradiol on proliferation. Thus, the sphingosine kinase pathway might be a mediator of a major part of estradiol activity on proliferation.

### Sphingosine‐1‐phosphate receptors are involved in hOB proliferation and differentiation

**Figure 2:**
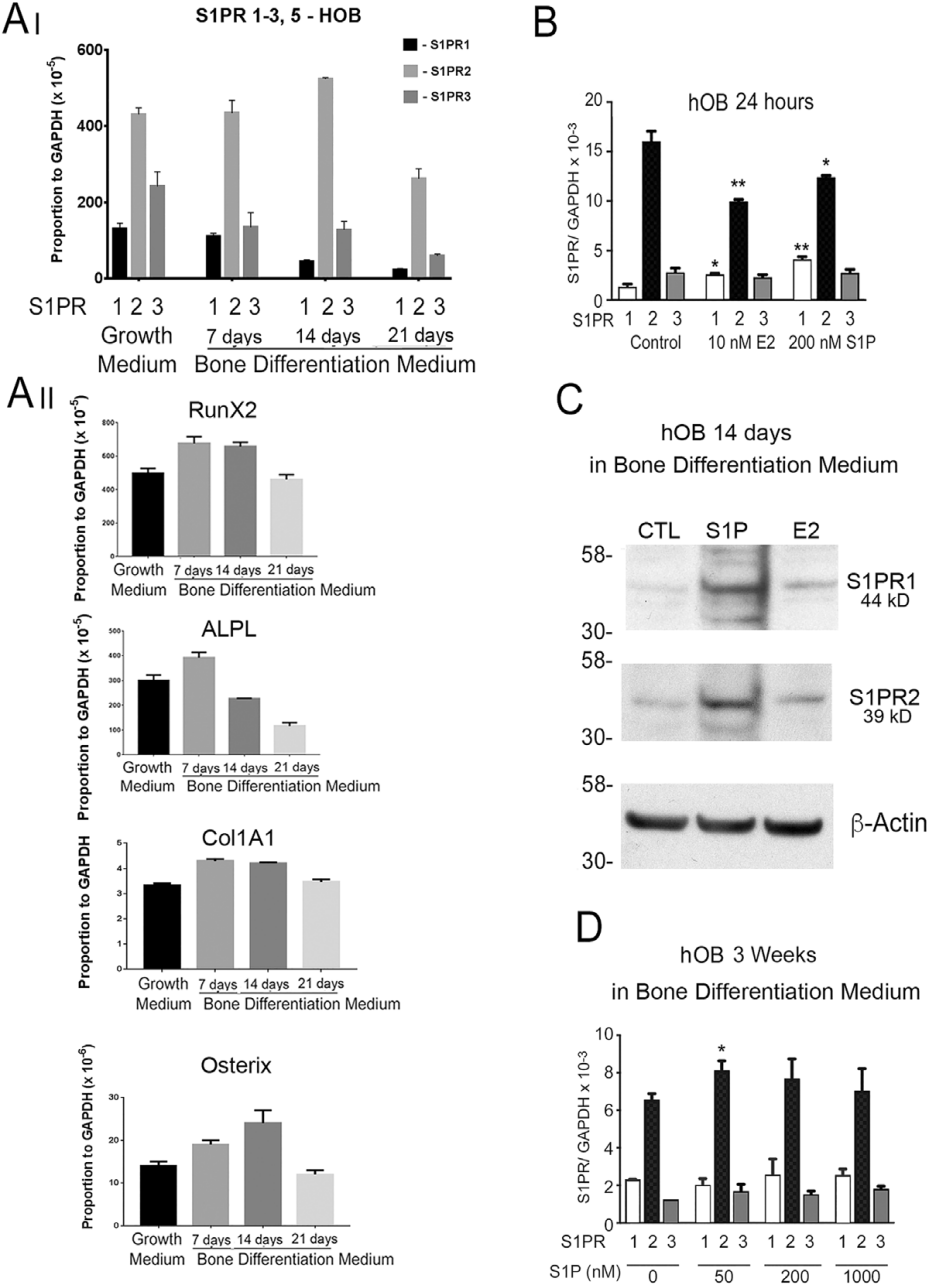
Expression of S1P receptors and osteoblast differentiation‐related mRNAs in human osteoblasts. (*A*) Analysis by real‐time PCR of the expression of S1P receptors 1, 2, and 3 (*A*i) in correspondence with osteoblast‐related mRNAs expression (*A*ii), including RUNX2, alkaline phosphatase, type I collagen, and osterix in undifferentiated hOB and after 7, 14, and 21 days in differentiation medium. (*B*) Effect of 24‐hour treatment with 10 nM estradiol (E2), or 200 nM S1P, on S1PR1‐3 in undifferentiated hOB. Both E2 and S1P increase S1PR1 mRNA expression, decrease S1PR2, and did not change S1PR3. (*C*) Both S1PR1 and S1PR2 protein production by hOB was increased with S1P treatment at 2 weeks of differentiation. (*D*) In late differentiation (3 weeks), effects of S1P on receptor mRNAs were limited to minor effects on S1PR2. Relative to control, *p < 0.05; **p < 0.01.

PCR data for sphingosine‐1‐phosphate receptors are shown in Fig. 2. During bone differentiation, S1PR2 was significantly increased (*p* < 0.05) at 14 days of differentiation and then decreased at 21 days (Fig. 2 *A*i) in correspondence with osteoblast‐related mRNA expression including RUNX2, alkaline phosphatase, type I collagen, and osterix (Fig. 2 *A*ii). S1PR1 and S1PR3 decreased progressively at 7, 14, and 21 days of differentiation (Fig. 2 *A*). Expression of S1PR4 and S1PR5 in hOB was negligible (not illustrated). Treatment with 10 nM E2 or 200 nM S1P, for 24 hours, decreased the mRNA expression for S1PR2 but increased S1PR1 mRNA. There was no significant effect on S1PR3 mRNA (Fig. 2 *B*). At 2 weeks of hOB differentiation, both S1PR1 and S1PR2 protein expression measured by Western blot analysis showed a definite increase with S1P treatment and a slight increase with estradiol (Fig. 2 *C*). At 3 weeks of osteoblast differentiation, the dose response of S1P receptors was further evaluated; S1PR2 increased slightly with 50 nM S1P, while the S1PR1 and S1PR3 were not changed (Fig. 2 *D*). These data, including the effect of the S1PR1 agonist SEW2871 on proliferation, suggested that S1PR1 and S1PR2 are involved in hOB differentiation and proliferation.

### S1P and E2 effects on undifferentiated hOB are mediated via activating the S1PR1/SPHK and MAP kinase activity

**Figure 3:**
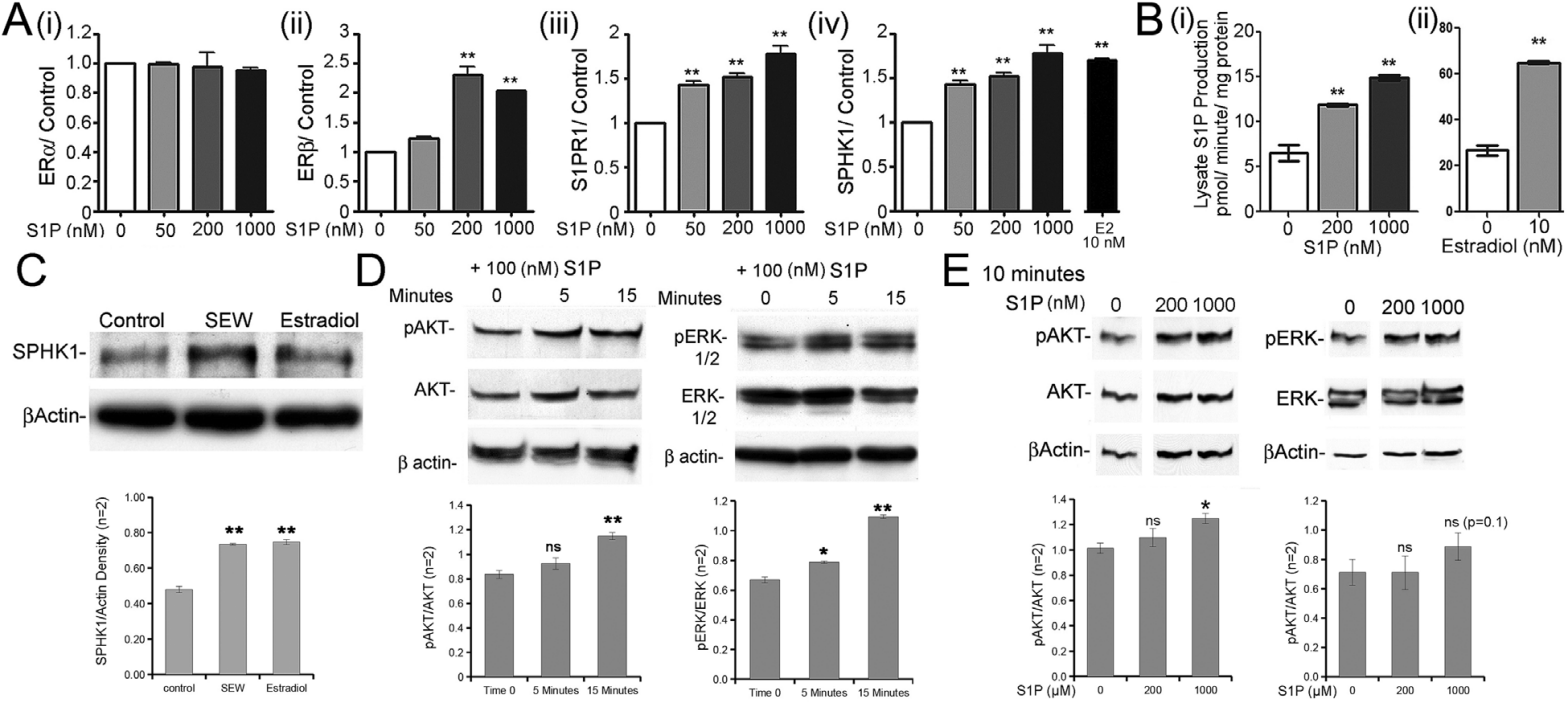
Short‐term effect of S1P or E2 on osteoblast estrogen receptors, SPHK activity, and MAPK pathways. (*A*) Effect of 24‐hour treatment with 1 μM S1P or 10 nM E2 on ER, S1PR, and SPHK1 mRNA expression. Note the concentration‐dependent increase in ERβ (ii), S1PR1 (iii), and SPHK1 (iv) mRNA expression. Expression of ERα mRNA (i) was unaffected. Effect of estradiol, 10 nM, on mRNAs for SPHK1 at 24 hours (iv). Real‐time PCR normalized to GAPDH, mean ± range, *n* = 2 (independent experiments with 2 to 4 replicates). *p < 0.05, **p < 0.01 versus control. (*B*) Effect of (i) 200 nM or 1000 nM S1P or (ii) 10 nM estradiol on sphingosine kinase activity. S1P stimulated the production of fluorescently labeled S1P, reflecting sphingosine kinase activity in osteoblast lysates, after treatment for 15 minutes with unlabeled S1P, pmoles/min/mg protein. Effects of estradiol were similar; separate experiments with separate controls. Mean ± SEM, *n* = 3 **p < 0.01 versus control. (*C*) On Western blot, SPHK1 protein production was increased at 24 hours by 10 nM estradiol, or the durable S1P receptor 1 agonist SEW2871, 1 μM. (*D*) Effect of S1P (100 nM) on AKT and ERK phosphorylation in h‐MSC in 5 and 15 minutes. S1P increased phospho‐AKT and phospho‐ERK at 15 minutes relative to AKT and ERK or to β‐actin. (*E*) S1P effect on AKT and ERK phosphorylation in hOB. S1P increased phospho‐AKT at 200 nM and 1000 nM S1P and slightly increase phospho‐ERK at 1000 nM. (*C*–*E*) Quantification of two ECL blot images normalized to equal total protein to eliminate artifacts of different loading or detection efficiency. *p < 0.05; **p < 0.01, ns (not significant).

We studied the dose‐response effect of S1P on ER, S1PR1, and SPHK1 mRNA expression in undifferentiated osteoblasts at 24 hours (Fig. 3 *A*). S1P did not alter ERα mRNA (Fig. 3 *A*i), but it upregulated ERβ (Fig. 3 *A*ii), S1PR1 (Fig. 3 *A*iii), and SPHK1 (Fig. 3 *A*iv) mRNA expression in a concentration‐related manner. At the same time point, 10 nM E2 upregulated SPHK1 mRNA (Fig. 3 *A*iv) and protein expression. By Western blot, either 10 nM E2 or 1 μM S1PR1 agonist SEW2871 increased SPHK protein at 24 hours (Fig. 3 *C*). These data are consistent with a mechanism where E2‐mediated proliferative effects, at least in part, occur through S1PR1/SPHK activity.

To determine the effect of S1P and E2 on SPHK activity in proliferating hOB, we measured S1P production by Fluorescent assay. At 15 minutes, S1P at both concentrations, 200 nM and 1000 nM, increased SPHK activity (Fig. 3 *B*i); E2 at 10 nM had a similar effect (Fig. 3 *B*ii), confirming involvement of S1P and SPHK in E2‐mediated proliferative effects.

Previously, we reported that E2 promotes osteoblast proliferation via activating the MAP kinase activity.^18^ In this study, we investigated the effect of S1P on the MAP kinase activity in osteoblast precursors (h‐MSC) and osteoblasts (hOB). S1P‐stimulated phospho‐AKT and phospho‐ERK at 15 minutes in h‐MSC are shown on Fig. 3 *D*. The concentration dependence of AKT and ERK phosphorylation at 10 minutes was studied in osteoblasts (hOB) at 0, 200, and 1000 nM S1P (Fig. 3 *E*), where increased phosphorylation of AKT was found at 200 and 1000 nM S1P and a trend to increased ERK phosphorylation at 1000 nM S1P, which did not reach significance. Protein expression and phosphorylation were quantified by densitometry analysis and normalized to the non‐phosphorylated form or to actin. These results suggest that osteoblasts’ response to the S1P, as in other cells, at least in part, involve ERK and possibly AKT phosphorylation.

### E2 and S1P promote osteoblast differentiation

**Figure 4:**
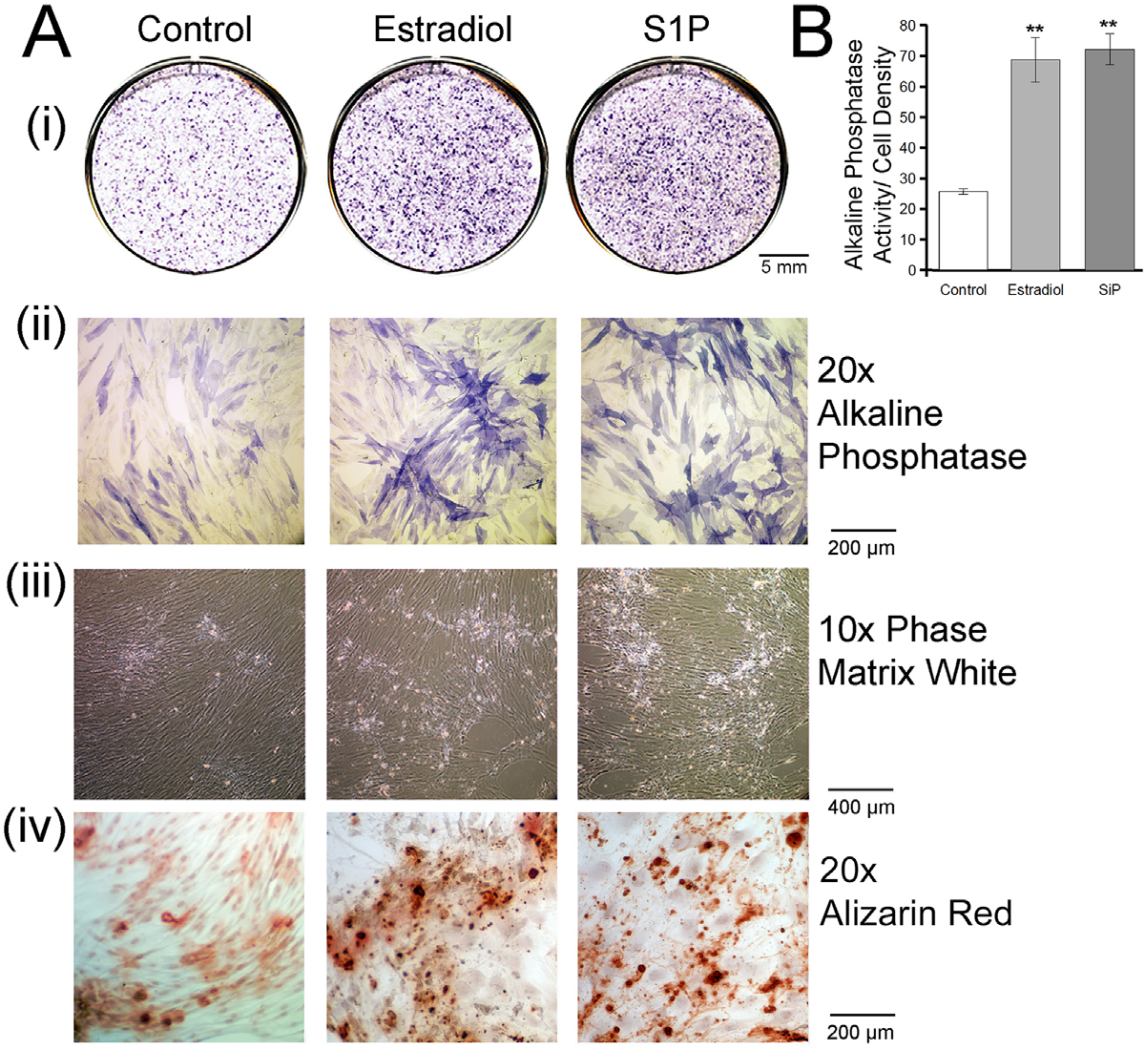
Estradiol (10 nM) or S1P (200 nM) promotes osteoblast differentiation. (*A*) (i) Alkaline phosphatase activity after treatment for 3 weeks is shown in whole 9 cm^2^ cultures. (ii) Cells photographed after staining with ALP at 20× magnification without phase. (iii) At low power, 10×, in phase, the dense developing tissue and collagen are revealed. (iv) Cells photographed after staining with alizarin red at 20× magnification. (*B*) ALP activity quantified by densitometry analysis and normalized to cell number. Both estradiol and S1P increase ALP activity compared with the control. Results are mean ± SEM, *n* = 4. **p < 0.01 versus control.

In bone differentiation medium, E2, 10 nM and S1P, 200 nM enhanced differentiation, indicated by increasing ALP, after 3‐week treatment. The expression ALP in the E2‐ and S1P‐treated group were higher than the control, as found in whole 9 cm^2^ cultures with alkaline phosphatase staining (Fig. 4 *A*i). ALP activity was quantified by densitometry analysis and normalized to cell number to confirm the in situ labeling. Either estradiol or S1P significantly (*p* < 0.01) increased ALP activity normalized to cell number (Fig. 4 *B*). The specificity of ALP expression for the cell surfaces is shown at 20× (Fig. 4 *A*ii). Increased matrix is shown at 10× in phase (Fig. 4 *A*iii). The increase of mineralization by S1P and E2 treatment was also confirmed using alizarin red (Fig. 4 *A*iv). These results indicate that both E2 and S1P promote osteoblast differentiation in vitro.

### Time course of S1P effects on osteoblast features, ERs, and S1P‐related genes

**Figure 5:**
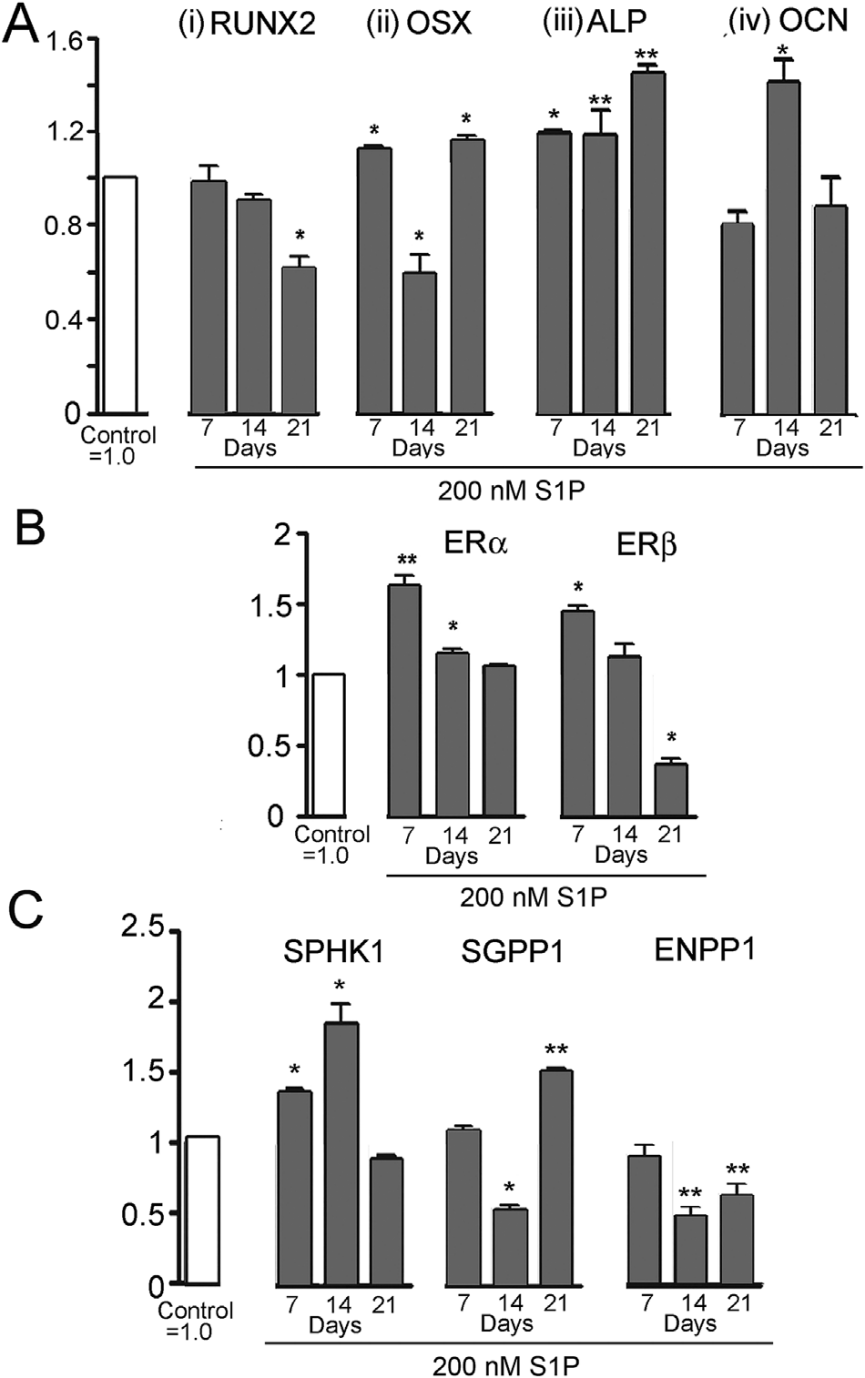
Time‐course effect of S1P on characteristic osteoblast, estrogen receptors, and S1P‐related mRNAs. All assays are real‐time PCR in duplicate, shown as mean ± range, and normalized in each case to matched controls = 1.0; *p < 0.05; **p < 0.01. (*A*) Time‐course effect of 200 nM S1P, with media changed every 3 days, on osteoblast‐related mRNAs. (*B*) Effect of long‐term treatment with 200 nM S1P on estrogen receptors (ERs). In contrast to effects at 24 hours (Fig. 2), where only ERβ increased, both ERα and ERβ mRNAs increased after 1 week in 200 nM S1P and dropped after 14 days, in keeping with cell maturation with reduced response to growth and differentiation factors. (*C*) The response to 200 nM S1P supplementation in media of S1P targets SPHK1, SGPP1, and ENPP1 mRNAs at 7, 14, and 21 day of hOB differentiation. The SPHK1, SGPP1 showed variable effects, but each was increased at either 14 or 21 days. ENPP1 was downregulated in osteoblast differentiation.

Further to explore the target that responds to S1P effect, we then evaluated by real‐time PCR the effect of S1P on the osteoblast‐related mRNA expression, including RUNX2, osterix (OSX), alkaline phosphatase (ALP), and osteocalcin (OCN) for 7 to 21 days (Fig. 5 *A*). OSX and ALP mRNA expression by hOB was significantly increased with 200 nM S1P treatment at 7 days of differentiation. There were decreases in RUNX2 (Fig. 5 *A*i) at 14 to 21 days, in keeping with maturation of cells (that is, full differentiation), whereas structural and mineralization‐related genes, including ALP (Fig. 5 *A*iii) and OCN (Fig. 5 *A*iv), were increased.

Because S1P showed the osteogenic effects comparable to estradiol, ER mRNAs were also determined (Fig. 5 *B*). In contrast to effects of 200 nM S1P at 24 hours (Fig. 3 *A*i, ii), where only ERβ mRNA expression were increased, 7 days of differentiated hOB treatment with 200 nM S1P significantly upregulated both ERα and ERβ mRNA. At 14 to 21 days, this effect was lost and both ERs decreased, in keeping with cell maturation with reduced response to growth and differentiation factors.

S1P targets, SPHK1, SGPP1, and ENPP1 mRNAs, were analyzed at 7 days of hOB differentiation after treatment with 200 nM S1P (Fig. 5 *C*). SPHK1 mRNA expression was significantly increased at 7 and 14 days, and SGPP1 was increased at 21 days. These results are consistent with maintenance of, and in some cases increased, S1P production and signaling during osteoblast differentiation. The signal transduction pathways that control ENPP1 expression are not well known, although from our work S1P does not upregulate ENPP1 mRNA in osteoblasts at 14 and 21 days of differentiation despite its association with osteoblast phosphate production.

### Inhibiting the sphingosine kinase (SKi) blocks the effect of E2 on the expression of many, but not all, osteoblast‐related mRNAs

**Figure 6:**
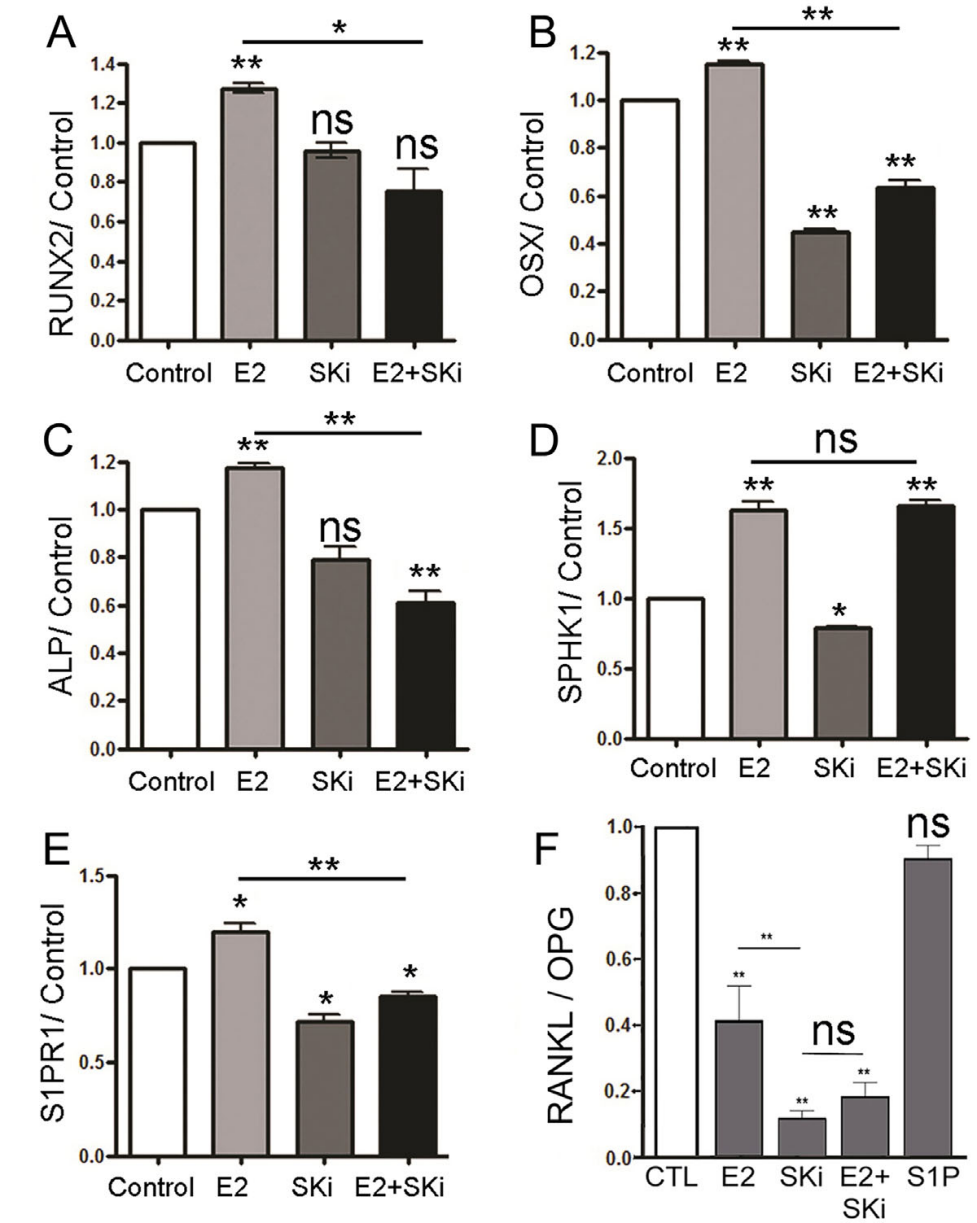
Effects of estrogen are reversed in many, but not all, cases by inhibiting the sphingosine kinase. Representative estrogen‐sensitive mRNAs in human osteoblasts are shown. For RUNX2 (*A*), OSX (*B*), ALP (*C*), and S1PR1 (*E*), mRNA expressions were induced by 10 nM estradiol at 7 days. A 10‐μM SKi treatment reduced mRNA expression, and the combination of estradiol and SKi mRNA expression remained less than the control. The SPHK1 (*D*) mRNA expression was decreased by the sphingosine kinase inhibitor SKi, but was induced by estradiol with or without SKi. (*F*) Estradiol and S1P have different effects on the RANKL‐OPG pathway. Data shown are for 7‐day treatment. The untreated control is defined as 1.0. RANKL/OPG mRNA expression ratio was decreased 60% to 80% by 5 nM estradiol treatment and further by the sphingosine kinase inhibitor (10 μMSKi) treatment; the combination of SKi and estradiol gave an intermediate result. On the other hand, RANKL/OPG was not reduced in 200 nM S1P. Media were changed at 3‐day intervals. Duplicate results ± range; *p* values are relative to control = 1.0 except when indicated by bars.

Fig. 6 shows representative estrogen‐induced mRNAs in hOB, after 7 days with or without 10 nM E2. In each case, RUNX2, osterix (OSX), alkaline phosphatase (ALP), sphingosine kinase‐1 (SPHK1), and the sphingosine‐1‐phosphate receptor‐1 (S1PR1) mRNAs are all induced significantly, 20% to 50%, by estradiol relative to controls. Addition of 10 μM SKi to controls in assays for RUNX2 and ALP mRNAs did not affect their expression; the other mRNAs were reduced significantly below the control. Further, the combination of SKi and E2 reduced mRNA expression relative to the control, except for sphingosine kinase 1 mRNA, which responded to estradiol with or without SKi.

The OPG/RANKL pathway, which is essential to balance of bone formation and resorption, was evaluated for the effects of S1P and E2. Although 10 nM E2 in differentiation medium for 2 weeks significantly reduced the ratio of RANKL to OPG, 200 nM S1P did not change the ratio of RANKL to OPG (Fig. 6 *F*). Addition of 10 μM SKi to the control hOB significantly reduced RANKL/OPG. Further, the combination of SKi and E2 reduced the ratio of RANKL to OPG mRNA expression relative to the control. These results confirm that many, but not all, estrogen effects on hOB differentiation are mediated, at least in part, via sphingosine kinase activity.

### Pathway analysis by whole‐genome mRNA expression

We studied undifferentiated human osteoblasts under control conditions or with addition of 10 nM estradiol or 200 nM S1P for 24 hours. Key pathway maps and changes in expression of individual proteins are shown in the Supplemental Data. In brief, osteoblast differentiation and cell adhesion pathways downstream of JAK kinases and signal transducer and activator of transcription (Stat1 and Stat5) intermediates were found (Supplemental Fig. S1). Additional cell adhesion and matrix maturation proteins were linked to Rho/Rac receptors with additional intracellular and cell surface targets, including actin and integrins identified (Supplemental Fig. S2). In accord with findings of S1P production, sphingosine kinase 1 activation downstream of Map kinases was indicated (Supplemental Fig. S3). An unexpected finding was strong activation by either E2 or S1P of superoxide dismutase 1 or 2 expression (Supplemental Fig. S4). Additional metabolic pathway links to Rho and Rac signaling and intermediate kinases included VEGF‐A expression (Supplemental Fig. S5).

## Discussion

Estradiol protects bone mass in a number of contexts and is known to suppress production of RANKL, which induces production of bone‐degrading osteoclasts.^19^ Estrogen signaling has genomic and nongenomic components, including estrogen signaling in bone.^13^ In non‐bone cells, including breast cancer, an important nongenomic signal downstream of estrogen is production of sphingosine‐1‐phosphate via SPHK1/2.^6^, ^20^ Our work demonstrates, using assays of fluorescently labeled S1P production, that estrogen or S1P, directly or indirectly, induce S1P production (Fig. 3 *B*); PCR for the sphingosine kinase 1, SPHK1, and Western blots were consistent. The time courses of S1P action in osteoblasts and the levels of S1P production in vivo are unknown; analyses of these are important future goals. Although S1P production in response to estrogen was not previously demonstrated in osteoblasts, it was suggested in recent work that S1P induces the osteoblast‐related transcription factor RunX2 in osteoblasts under some conditions^1^ and promotes osteoblastic differentiation in pluripotent cells (C3H10T1/2).^21^ Our work on hOB did not show similar RunX2 induction, although it is clear that cells respond quite differently when studied under different conditions.

Sphingosine‐1‐phosphate signaling is complex and it is often difficult to separate functions of individual receptors. Overall, S1PR1 mediates chemotaxis toward S1P via a G_i_ Rac, whereas S1PR2 inhibits migration via G_12/13_ Rho, which inhibits Rac.^22^ Further, S1PR1 stimulates proliferation via G_i_‐mediated pathways, including PI3K/Akt and Erk, whereas S1PR2 can inhibit proliferation by Rho/Rho kinase‐dependent Akt inhibition, reviewed in Takuwa and colleagues.^22^ More specifically for MSC and bone, S1PR2 regulates MSC proliferation, migration, and stability via increases in pluripotency factors that reduce osteoblast or adipocyte differentiation.^23^ S1PR1, S1PR2, and S1PR3 are expressed by osteoblasts (Fig. 2). Importantly, S1PR1 expression in undifferentiated cells responds to S1P in a concentration‐dependent manner (Fig. 3 *A*iii). This is potentially important because the S1PR1 agonist SEW2871^16^ replicated half of estrogen effects on osteoblast proliferation (Fig. 1 *A*). In other work, S1PR1 signaling modulates PDGF‐induced motility.^24^ Additional work on osteoblasts and osteoblast‐like cells is consistent with S1PR1 and S1PR2 regulation of osteoblast maturation by kinase cascades,^1^ although it is unclear the relation of maintenance of undifferentiated MSC features to osteoblast maturation.

Our work is consistent with involvement of Map kinases in S1P regulation (Fig. 3 and Supplemental Figs. S1–5), although kinase activation by S1PR3 was not strong. Studies in MSC maturation also suggest that S1PR1/2 coordinate expression of kinases including Akt,^25^ pathways also supported by genomewide expression data (Supplemental Fig. S2). In other cells including breast cancer, S1P signals in part by MAP kinase activation,^26^ and in our work osteoblast response to S1P is consistent with this. The induction of MAP kinase leads to translocation of SPHK1 from the cytosol to the plasma membrane, resulting in S1P production.^27^ It remains to be seen how related pathways are affected in osteoblasts and comparisons with other cells. This will determine the therapeutic potential for S1P activation and inhibition in bone relative to other organs. Further, there is evidence that S1P via S1PR3 is physiologically functional in mouse marrow in commitment of stem cell precursors,^28^ arguing that the S1P system in bone needs to be further analyzed.

There was overlap in effects of S1P and estrogen in human osteoblasts in promoting short‐term cell proliferation (Fig. 1). That this overlap is due to functional biochemical linking is suggested by partial replication of the effects of S1P by the S1PR1 analog SEW2871 and that the S1P antagonist SKi opposed the effects of estradiol. The effects of S1P and estradiol on osteoblast maturation and bone formation also had strong similarity (Fig. 4). Estradiol at 10 nM upregulated the sphingosine kinase‐1 at 24 hours, consistent with effects of estradiol on S1P production (Fig. 3). It will be important, however, to determine the half‐life of S1P physiologically because human osteoblasts also express the S1P lyase, which irreversibly inactivates S1P (not illustrated). The expression and activity of sphingosine phosphatases are likely also to have important effects on S1P levels, even though their effects are reversible. Our data show that sphingosine‐1‐phosphate phosphatase 1, SGPP1, is expressed in osteoblasts and regulated by S1P (Fig. 5 *C*). Elements of the pathway including the lyase and the SPHKs are potential therapeutic targets in bone and other organs.^29^ In osteoblast differentiation, S1P reduced bone‐regulating estrogen receptors at late times, in keeping with cell maturation. Mature bone cells (osteocytes) have reduced response to estrogen, losing ERα and ERβ.^30^

The concentrations of S1P used in cell biology represent the range of typical and high‐serum concentrations of 0.2 to 1 μM; further work will be needed to determine what part of this range is relevant. In this regard, however, serum blood concentrations of S1P are often 0.2 μM in humans and 0.7 μM in mice and including red blood cell S1P concentrations may exceed 2 μM, reviewed by Thuy and colleagues.^31^ Serum albumin facilitates release of S1P, without binding it.^31^ In bone, little is known about actual S1P concentrations, although our data indicate that bone cells make significant amounts of S1P (Fig. 3 *B*).

Despite the clear overlap in S1P and estrogen signaling, some genes respond to estradiol but not to S1P. A good example of this is osteoprotegerin production relative to RANKL (Fig. 6). Another example is the sphingosine kinase 1 mRNA, which is upregulated by S1P at high concentrations or by 10 nM estradiol (Fig. 3 *A*, *C*), but where the estradiol effect is unchanged by adding the sphingosine kinase inhibitor SKi (Fig. 6 *D*). Determining tissue expression of S1P under varying conditions will be a challenging target of further work and will be important to evaluate the role of this new pathway in the regulation of bone differentiation. Measurements of S1P in serum indicate that it circulates at concentrations of 500 to 1000 nM,^32^ albeit significantly protein bound and in some circumstances released by platelet activation. Carriers of S1P include albumin and apolipoprotein M; in serum, S1P is important in vascular homeostasis and immune cell response.^33^, ^34^ There are precedents for osteoblast‐derived S1P as potentially protecting cancer proliferation,^35^ but at present physiological levels of S1P in bone and regulation of its production in vivo are not well characterized.

We describe the function in bone cells of sphingosine‐1‐phosphate production in response to estradiol via the SPHK, with secondary effects via S1P and sphingosine‐1‐phosphate receptors 1 and 2 that mediate, at least in part, estrogen effects on bone. S1P production in osteoblasts is stimulated by estradiol at high but physiological concentrations. There are many areas of overlap between S1P‐mediated and estradiol‐mediated effects on osteoblast maturation, although some differences exist, including that estradiol, but not S1P, regulates production of osteoprotegerin relative to RANKL. Differences in signaling might be important in physiological response of bone to S1P inhibitors or activators as potential modulators of bone formation.

## Disclosures

All authors state that they have no conflicts of interest.

## Supporting information

Supporting Data S1.
