# Supplementary material for: Sphingosine‐1‐Phosphate Modulates the Effect of Estrogen in Human Osteoblasts
Source: JBMR Plus. 2018 Mar 15;2(4):217–26. doi: 10.1002/jbm4.10037 (PMC6095197; doi:10.1002/jbm4.10037)

**Supplemental Data**

Maps of 212 established signaling pathway by by MetaCore (St Joseph, MI) using data from control, S1P, and estrogen treated cells from Affymetrics chip analysis as described in Methods. Briefly, Osteoblast mRNA in cells were harvested from control cells or after 18 hour treatments with S1P, 1 µM, or E2, 10 nM. Presence of transcripts and differences between treatments were determined from the signal and variation of 20 assay replicates. Analysis excluded genes not expressed with p > 0.05. Differences with p < 0.002 between conditions are included in analysis.

The main differences described in each supplemental figure are structural proteins, enzymes, and receptors. Expression of many kinases that are elements of the pathways are not changed, an expected result since these proteins are regulated by phosphorylation, for the most part, rather than by changes in protein synthesis. Exceptions included Akt expression, with modest but significant changes.

Elements found in several pathways may be described only once, for brevity. For numerical data below, in all cases the highest expression with S1P or E2 has p < 0.02 relative to control. In a few cases, responses to S1P and E2 differ.

**I. Osteoblast differentiation and cell adhesion pathways downstream of JAK kinases and signal transducer and activator of transcription (Stat1 and Stat5) intermediates.**

Interesting results include changes in pathways identified in oteoblast differentiation including fibronectin, integrins, and Jak2. Upstream are increases in pyruvate kinase **(**PKM2) and three Stat proteins, Stat1, Stat3, and Stat6. There are modest changes in Akt and Map kinases.

**Fibronectin** 1 – Control – 35014; 2 - S1P – 53388; 3 - E2 - 43050.

**Integrin Beta 1** 1 – Control – 10932; 2 - S1P – 14152; 3 - E2 - 14954.

**JAK1** 1 – Control – 1157; 2 - S1P – 1477; 3 - E2 - 1558.

**JAK2** 1 – Control – 560; 2 - S1P – 730; 3 - E2 - 801.

**STAT1** 1 – Control - 20005; 2 - S1P - 24414; 3 - E2 - 24302.

**STAT3** 1 – Control - 1318; 2 - S1P - 2138; 3 - E2 - 2457.

**STAT6** 1 – Control - 3334; 2 - S1P - 4403; 3 - E2 - 4314.

**PKM2**  1 – Control - 12232; 2 - S1P - 16676; 3 - E2 - 24971.

**AKT** 1 – Control - 1264; 2 - S1P - 1849; 3 - E2 - 2275.

**MEK3** 1 – Control - 2950; 2 - S1P - 2908; 3 - E2 - 3589.

**MEK7** 1 – Control - 374; 2 - S1P - 427; 3 - E2 - 764.


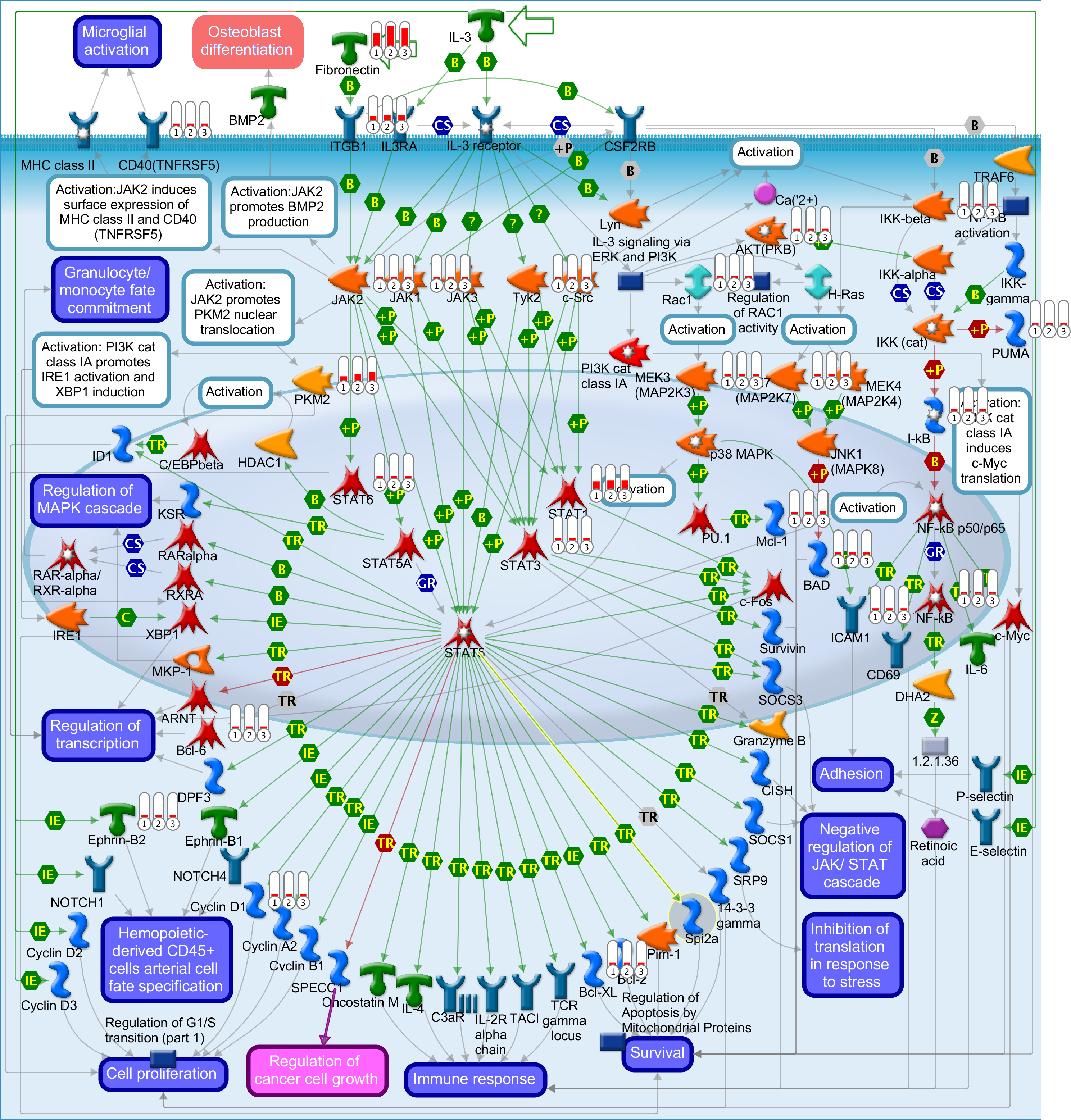


**II. Cell adhesion (i) and matrix maturation (ii) proteins were linked to Rho/Rac receptors with additional intracellular and cell surface targets including actin and integrins.**

Interesting results from this pathway include similar increases with S1P or estrogen in fibronectin and integrins, shown above in pathway I, in Type I collagen and the intracellular cytoskeletal actin. Root signaling proteins including RhoA, Rac1 and Stat1 are increased **(i)**; these are also found in other pathways. Extracellular proteins **(ii)** include changes in osteonectin, in tissue inhibitors of metalloproteinases, and collagens III and IV. Collagen III is found in many connective tissues including bone. Type IV collagen is not a bone collagen, but found in basement membranes and is associated with endothelial cell differentiation. This may reflect that hOB differentiation includes cells other than osteoblasts *per se*, since bone is an organ not a single cell type. TIMP, tissue inhibitor of metalloproteinases.

**Rho A** 1 – Control - 17357; 2 - S1P - 18762; 3 - E2 - 21230.

**Rac1** 1 – Control - 4206; 2 - S1P - 4504; 3 - E2 - 5108.

**Actin** 1 – Control - 21482; 2 - S1P - 27426; 3 - E2 - 29179.

**Thrombospondin** 1 – Control - 2602; 2 - S1P - 4099; 3 - E2 - 7381.

**TIMP 1**  1 – Control - 34578; 2 - S1P - 55909; 3 - E2 - 44169.

**TIMP 2** 1 – Control - 2883; 2 - S1P - 3823; 3 - E2 - 4143.

**TIMP 3** 1 – Control - 3643; 2 - S1P - 4917; 3 - E2 - 5114.

**Osteonectin** 1 – Control - 25772; 2 - S1P - 37125; 3 - E2 - 31824.

**Collagen I** 1 – Control - 31611; 2 - S1P - 45657; 3 - E2 - 38944.

**Collagen III**  1 – Control -29369; 2 - S1P - 42919; 3 - E2 - 35731.

**Collagen IV** 1– Control - 5403; 2 - S1P - 8069; 3 - E2 - 10275.


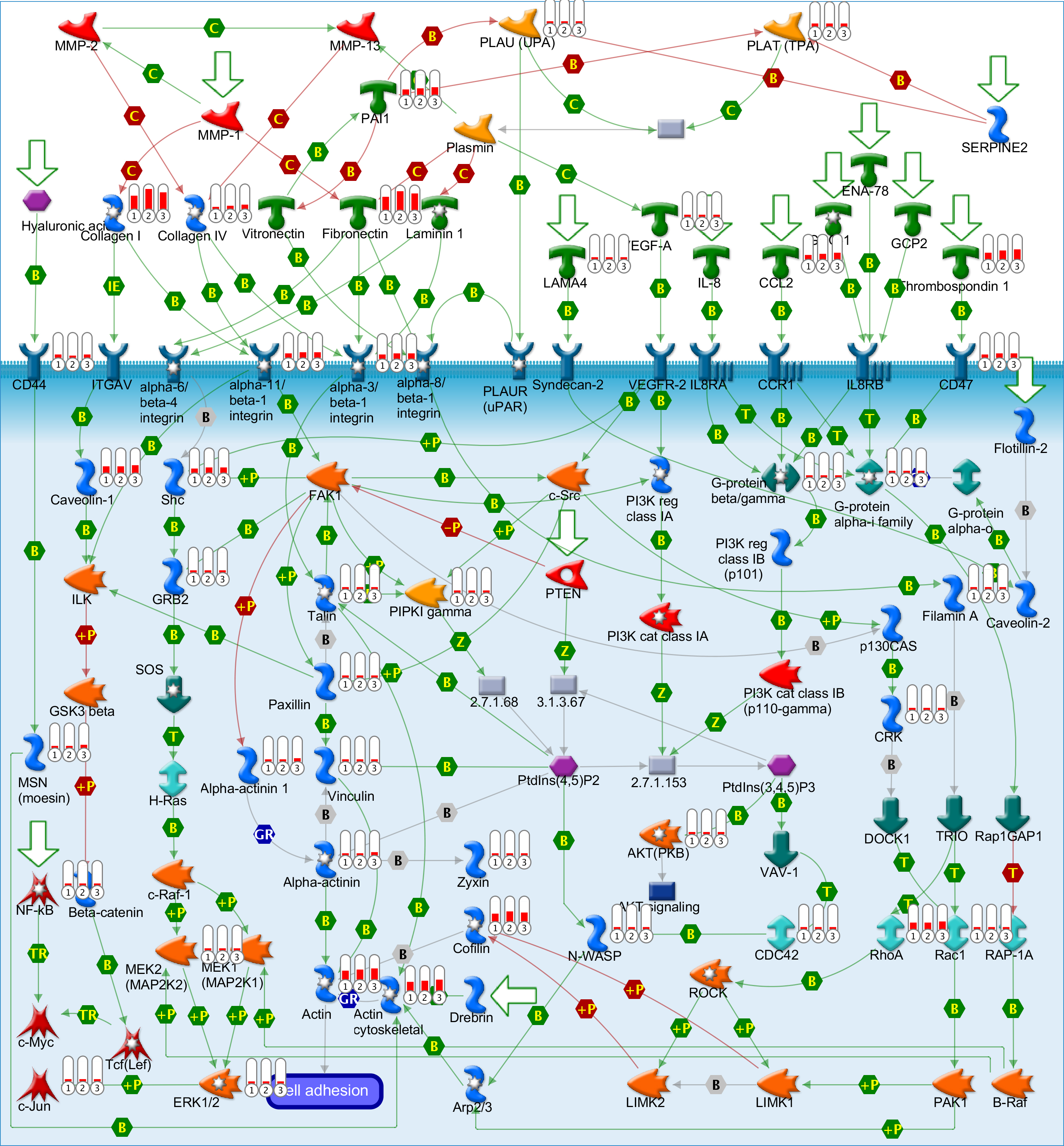


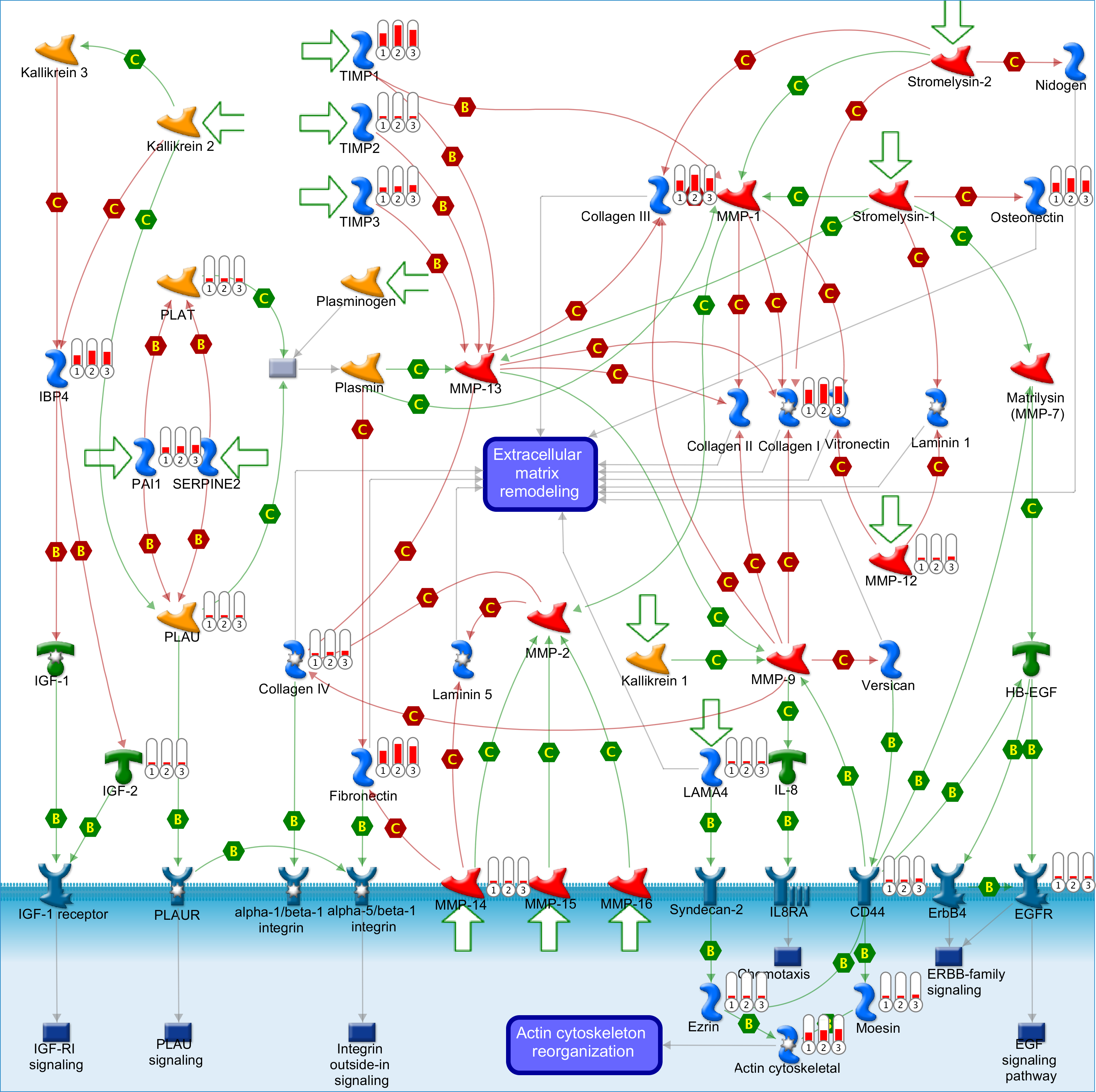


**III. Sphingosine kinase 1 (SPHK1) activation downstream of Map kinases.**

This interesting pathway map links elements including sphingosine kinase I, Stat1/3, and regulators of apoptosis including Bcl-XL, Mcl-1 and Shc1, and production of the essential protein VEGF-A which is required for production of vascular cells. SPHK1.

**SPHK1** 1 – Control – 1805; 2 - S1P – 2086; 3 - E2 - 1812.

**STAT1** 1 – Control – 20005; 2 - S1P – 24414; 3 - E2 - 24302.

**STAT3** 1 – Control – 1318; 2 - S1P – 2138; 3 - E2 - 2457.

**Bcl-XL** 1 – Control – 320; 2 - S1P – 1038; 3 - E2 - 1302.

**Mcl-1** 1 – Control – 3626; 2 - S1P – 4163; 3 - E2 - 5656.

**Shc** 1 – Control - 10182, 2 - S1P - 13922, 3 - E2 - 13303.

**VEGF-A** 1 – Control - 1281, 2 - S1P - 1602, 3 - E2 – 1539.


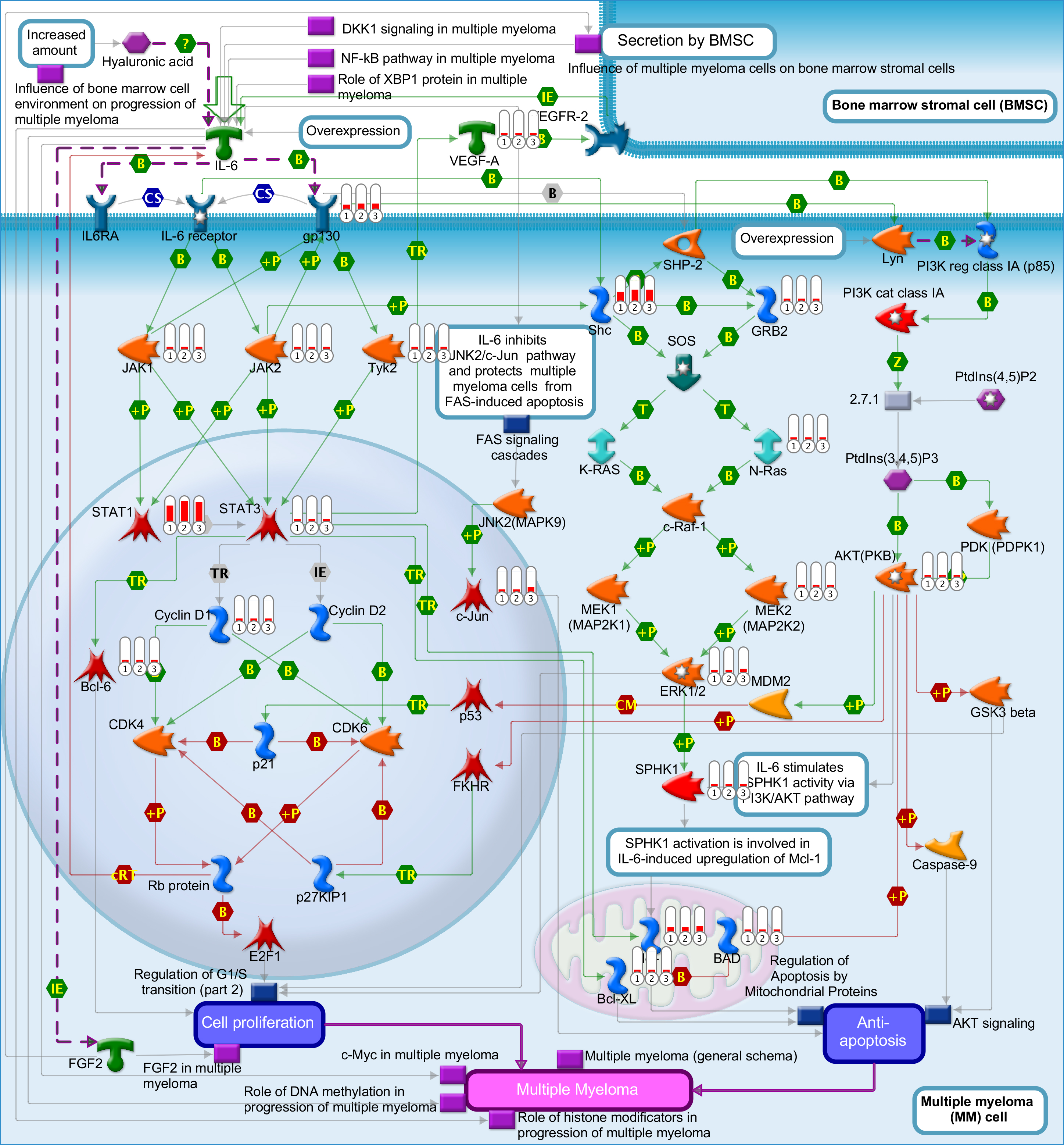


**IV. Increase of superoxide dismutase (SOD) 1 and 2 expressions.**

This pathway links strong increases, essentially unprecedented, in superoxide dismutases 1 and 2 to intermediate signals described in previous pathways. While SOD in osteoblasts is previously described, and effects of estrogen as well, the link to S1P is novel as far as we can determine.

**SOD1** 1 – Control – 9020; 2 - S1P – 10063; 3 - E2 - 11639.

**SOD2** 1 – Control -16176; 2 - S1P – 21276; 3 - E2 - 19092.


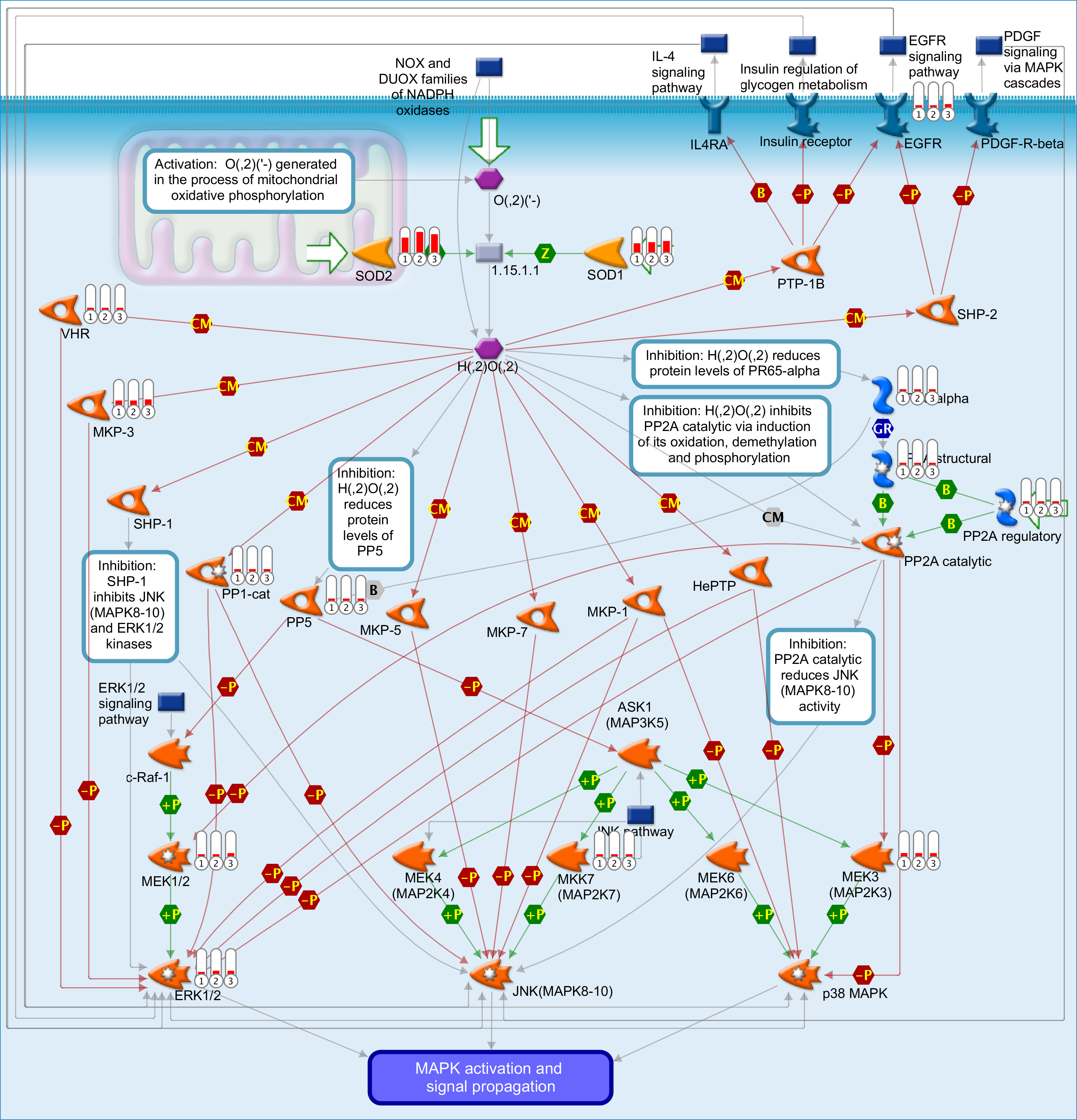


**V. Additional metabolic pathways that link Rho and Rac, and intermediate kinases signaling to VEGF-A expression and signaling.**

This pathway map links additional proteins to VEGF-A expression and signaling. Novel proteins in this pathway include chemokine (C-C motif) ligand 2 (CCL2), I-kB, calmodulin, and HSP90. Other kinases and elements described above are parts of the pathway but these, including VEGF-A, are for brevity not repeated.

**CCL2** 1 – Control – 12251; 2 - S1P – 14963; 3 - E2 - 17760.

**I-kB** 1 – Control – 3698; 2 - S1P – 6089; 3 - E2 - 5033.

**Calmodulin** 1 – Control – 15156; 2 - S1P – 18047; 3 - E2 - 19323.

**HSP90** 1 – Control – 8552; 2 - S1P – 9320; 3 - E2 - 12347.


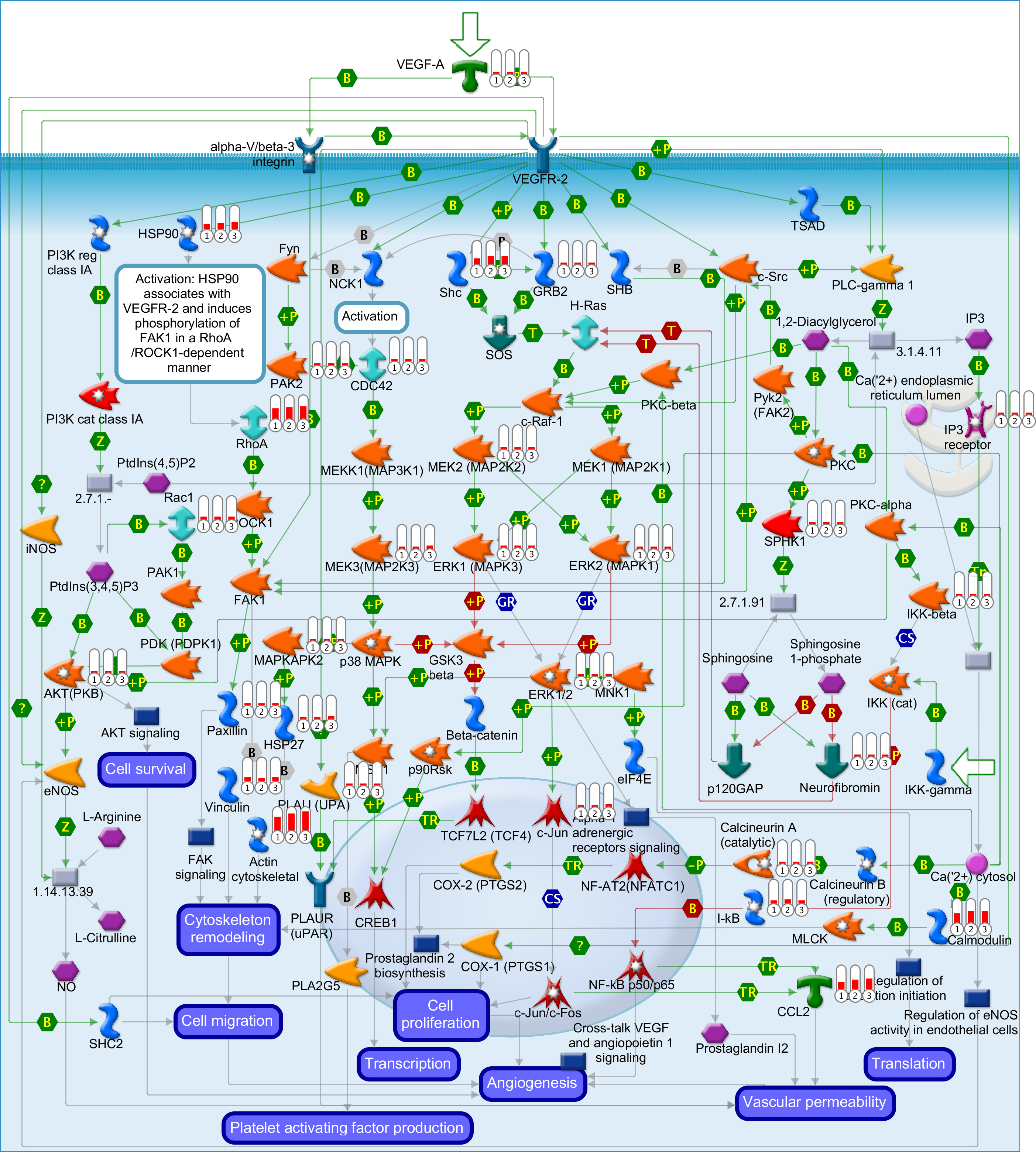

Supplement: Supplementary file 1 — Supporting Data S1. [file JBM4-2-217-s001.doc]
